# Arecoline Induces Testicular Injury and Impairs Spermatogenesis in Rats Through Notch Signaling-Mediated Oxidative Stress

**DOI:** 10.3390/ijms27219728

**Published:** 2026-10-29

**Authors:** Yan-Qi Yang, Wen-Xing Li, Pan Ge, Mo-Qi Lv, Ying Sun, Qing Chen, Jian Zhang, Rui-Fang Sun, Dang-Xia Zhou, Ming Liu

**Affiliations:** 1 Department of Obstetrics and Gynecoloy, The Second Affiliated Hospital of Xi’an Jiaotong University, Xi’an 710004, China; 2 Department of Pathology, School of Basic Medical Sciences, Health Science Center, Xi’an Jiaotong University, Xi’an 710061, China; 3 Department of Neurosurgery, Xi’an Jiaotong University Medical College First Affiliated Hospital, Xi’an 710061, China

**Keywords:** arecoline, notch signaling pathway, oxidative stress, reproductive toxicology, spermatogenesis

## Abstract

Millions of people chew areca nut to boost work efficiency and relieve stress. Areca nut contains arecoline, which adversely affects reproductive function, although the underlying molecular mechanisms remain unclear. Herein, we evaluated arecoline-mediated reproductive toxicity using Sprague–Dawley (SD) rat models and an in vitro GC-1 cell model. Molecular docking, quantitative reverse transcription PCR (qRT-PCR), Western blot, cell-based assays, and functional rescue experiments were used to investigate the mechanisms involved. Dose-dependent spermatogenic damage was observed in male rats receiving 50, 100 and 200 mg/kg arecoline, with reduced sperm counts and quality, higher testicular MDA and lower activity of antioxidant enzymes superoxide dismutase (SOD), catalase (CAT), and glutathione peroxidase (GPx). Molecular docking predicted a possible interaction between arecoline and Notch1. qRT-PCR and Western blot analyses showed that the expression of Notch1 and its ligands, Jagged-1 and Dll-1, was downregulated in testicular tissues and GC-1 cells following arecoline exposure. Notch1 expression was strongly positively correlated with seminiferous tubule diameter and seminiferous epithelium height. In vitro, arecoline induced ROS accumulation, inhibited proliferation and promoted apoptosis in GC-1 cells. Rescue experiments showed that the Notch activator valproic acid, used to enhance Notch signaling, alleviated arecoline-induced toxicity, whereas Notch inhibitor FLI-06 worsened cell injury. These findings support a role for altered Notch signaling and oxidative stress in arecoline-induced spermatogenic injury and provide insight into the molecular mechanisms of its reproductive toxicity.

## 1. Introduction

Infertility constitutes a major global reproductive-health burden in contemporary society [1,2]. Approximately 8–12% of couples worldwide experience infertility, with male-factor infertility contributing to roughly 50% of these cases [3,4]. A substantial proportion of male infertility arises from disrupted spermatogenesis, which manifests as reduced sperm concentration, impaired motility or abnormal sperm morphology. Semen quality therefore serves as a pivotal indicator for evaluating male reproductive health [5]. Studies in Chinese [6] and Western populations [7] have consistently documented a temporal decline in sperm quality over recent decades. The pronounced deterioration in semen quality is more likely attributable to lifestyle-related risk factors and social determinants [8,9] rather than genetic causes. Among such modifiable lifestyle exposures, areca-nut chewing represents a prevalent yet underappreciated risk factor for male reproductive health.

Areca nut is the fourth-most frequently consumed psychoactive agent globally, after tobacco, alcohol, and caffeine [10]. An estimated approximately 600 million individuals of all ages worldwide chew areca nut [11], most of whom reside in Central and South Asia, Southeast Asia, and Melanesia, including countries such as India, Pakistan, Bangladesh, Myanmar, Malaysia, Taiwan, and other countries [12,13]. The International Agency for Research on Cancer (IARC) classified areca nut as a species carcinogenic to humans and assigning it to a Group 1 human carcinogen in 2004 [14], whereas its main principal arecoline was classified separately as possibly carcinogenic to humans (Group 2B) in 2020 [15]. However, research on areca nut and arecoline has thus far focused almost exclusively on neuropsychiatric disorders, respiratory diseases, and malignancies of the upper digestive tract [16,17,18,19,20], whereas their effects on the reproductive system remain poorly characterized [21].

Areca nut contains several alkaloids—arecoline, arecaidine, guvacine and guvacoline—among which arecoline is the most abundant and pharmacologically potent [22]. Arecoline is a small lipophilic pyridine alkaloid (MW 155.19 g/mol) that occurs as an oily liquid at room temperature and exhibits low aqueous solubility [15]; nonetheless, it is readily absorbed and can be detected in blood, urine, saliva, hair and breast milk following areca-nut chewing [23,24]. In the present study, we focused on arecoline rather than on the whole areca-nut extract for three reasons: Firstly, arecoline is the principal bioactive constituent of areca nut and accounts for the majority of its pharmacological and toxicological effects [25]. Secondly, the whole areca-nut extract is a complex mixture of alkaloids, polyphenols, tannins, and flavonoids, and the reproductive phenotypes observed after exposure to the crude extract cannot be unambiguously attributed to any single component. Thirdly, the use of a chemically defined compound allows reproducible dose–response characterization and mechanistic dissection, which is essential for elucidating the mechanism by which arecoline disrupts spermatogenesis. We acknowledge, however, that this reductionist single-compound design does not reproduce human chewing, in which users are exposed by the oral route to the whole mixture; our objective is to isolate the contribution and the mechanism of the principal alkaloid.

There have been initial investigations into the male reproductive toxicity induced by both areca nut and arecoline [26]. For instance, Kiong Er et al. assessed the effect of varying arecoline concentrations of arecoline on sperm motility in the semen sample of 30 volunteers, reporting that arecoline exposure markedly diminished key sperm-motility parameters, including motility, average path velocity, curvilinear velocity, straight-line velocity and linearity [27]. The same study was also the first to demonstrate that arecoline upregulates cyclooxygenase-2 (COX-2) expression in human sperm in a dose-dependent manner, suggesting that an inflammatory response contributes to damage of the structures responsible for flagellar motion [27]. Furthermore, Yuan et al. substantiated that three major alkaloids derived from areca nut (arecoline, arecaidine, and guvacine) all inhibit sperm motility, with arecoline producing the most potent inhibitory effect on human semen [28]. Consistent with these in vitro observations, Wu and colleagues demonstrated that rats administered areca-nut extract displayed reproductive impairment, characterized by aberrant sperm-head morphology, reduced sperm count, and reduced sperm motility [29]. Additionally, Rahman et al. reported that 50 mg/kg areca-nut extract disrupted the morphology of the testicular tissue in rats, in which sperm count and the number of progressively motile sperm were lower than those in the control group, and treated male rats failed to impregnate female counterparts [30]. An in vivo study in mice that used purified arecoline likewise reported decreased body and testis weight, reduced sperm count, motility and proportion of morphologically normal sperm, accompanied by a decline in reduced glutathione (GSH), glutathione-S-transferase (GST), superoxide dismutase (SOD) and catalase (CAT) [31]. Collectively, these in vitro and in vivo findings demonstrate the reproductive toxic phenotypes induced by areca nut/arecoline. However, current understanding remains largely restricted to phenotypic correlations, with insufficient mechanistic explanations. Most in vivo evidence was obtained using whole areca nut or crude extracts. Even those studies employing purified arecoline only adopted a single dose; therefore, the independent toxic contribution of arecoline and its dose–response relationship has not been well established. Furthermore, most readouts were limited to descriptive observations such as sperm parameters and testicular morphological alterations, and the molecular targets of arecoline within spermatogenic cells remain unidentified. In addition, the signaling pathway linking arecoline exposure to spermatogenic impairment has not been delineated. Building on these critical knowledge gaps outlined above, the present study was performed to further explore the detailed mechanisms of arecoline-induced spermatogenic injury.

On the one hand, arecoline-induced oxidative stress is considered the primary contributor to its toxicity, which is associated with the excessive production of reactive oxygen species (ROS), imbalance of the antioxidant defense system, destruction of cellular substructures, and regulated cell death [32]. On the other hand, spermatogenesis is intrinsically vulnerable to redox imbalance: spermatogenic cells and spermatozoa are rich in polyunsaturated fatty acids and possess limited antioxidant reserve, whereas physiological levels of ROS are required for spermatogonial proliferation [33], capacitation and the acrosome reaction; once ROS exceed antioxidant capacity, lipid peroxidation, DNA damage and germ-cell apoptosis ensue [34]. Because oxidative stress is also the converging downstream node shared by most toxicants that impair spermatogenesis, it provides both a quantitative mechanistic readout and a tractable endpoint for pharmacological intervention.

Oxidative stress therefore represents a promising starting point for investigating how arecoline damages spermatogenic cells. Nevertheless, the mechanisms by which arecoline modulates intracellular signaling cascades to alter oxidative stress, especially within spermatogenic cells, remain incompletely elucidated. As a highly conserved signaling axis, the Notch pathway exerts critical functions in the development and pathological progression of multicellular organisms [35,36,37]. Notably, existing studies have shown that the Notch signaling pathway also serves as an essential regulator of spermatogenesis, governing the self-renewal and differentiation of spermatogonial cells and the regulation of the spermatogenic cycle [38,39]. Of particular relevance, recent studies have demonstrated that the Notch signaling pathway can regulate the cellular response to oxidative damage [40,41], which positions it upstream of ROS generation. Despite these prior findings, it remains unclarified whether arecoline exposure perturbs oxidative homeostasis in spermatogonial cells via the Notch signaling pathway and consequently induces spermatogenic impairment.

We therefore put forward the core hypothesis of this study: arecoline may interact with Notch1 and downregulate its expression. Reduced Notch1 expression suppresses the Notch signaling pathway, elevates ROS production, and thereby induces apoptosis and inhibits proliferation in spermatogenic cells. This study sought to investigate the impacts of graded arecoline doses of arecoline (50 mg/kg, 100 mg/kg and 200 mg/kg) on the male reproductive system in rats. Sperm quality, testicular tissue morphology, oxidative-stress biomarkers, the expression of Notch-signaling-related genes, and proliferation- and apoptosis-related genes were evaluated to determine whether verify arecoline induces reproductive disorders in a dose-dependent manner and to decipher its underlying molecular mechanism. In addition, an in vitro model employing arecoline-exposed spermatogonial cell line was characterized, laying a foundation for further dissection of the mechanisms driving arecoline-induced reproductive toxicity.

To the best of our knowledge, this study is the first to define an in vivo dose–response relationship for purified arecoline in spermatogenesis using a rat model complemented by an in vitro spermatogonial cell model—thereby separating the contribution of arecoline itself from that of the crude extract. Beyond this phenotypic characterization, molecular docking identified Notch1 as a candidate target of arecoline, and the extent of Notch1 downregulation was quantitatively related to seminiferous tubule diameter and seminiferous epithelium height. Causality was then established by combining bidirectional pharmacological interventions (the Notch activator valproic acid and the Notch inhibitor FLI-06) with Notch1 knockdown via si-Notch1; the knockdown reproduced the arecoline phenotype in the absence of the compound, demonstrating that Notch1 acts upstream of ROS generation, germ-cell apoptosis, and proliferation arrest. Our findings may reveal the toxic effects of arecoline on the male reproductive system and offer novel mechanistic insights into arecoline-associated health risks in mammals.

## 2. Results

### 2.1. General Systemic Status, Body and Testicular Weight

Longitudinal monitoring of body weight was performed throughout the 30-day exposure period. No mortality was observed in any experimental group. General systemic observations were recorded daily following arecoline administration. Transient tremor and seizure-like behaviors were detected shortly after each intraperitoneal injection of arecoline. Arecoline is a cholinergic agonist; these acute transient responses are attributed to the temporary activation of cholinergic signaling immediately after injection, and the symptoms spontaneously resolved within 1–2 min. At day 0, day 10 and day 20, there were no statistically significant differences in body weight among the control, 50 mg/kg, 100 mg/kg and 200 mg/kg groups. A significant reduction in body weight was observed in the 200 mg/kg group at day 21, and the difference became more prominent at day 30, reflecting slowed body weight gain at the later stage of exposure. Arecoline administration at 50 mg/kg and 100 mg/kg did not significantly alter rat body weight compared with that of the corresponding control animals (Figure S2, Table 1). In addition, testicular weights did not differ significantly between any arecoline-treated group and the corresponding control group (Table 1).

### 2.2. Arecoline Induces Dose-Dependent Testicular Damage in Rats

**Figure 1:**
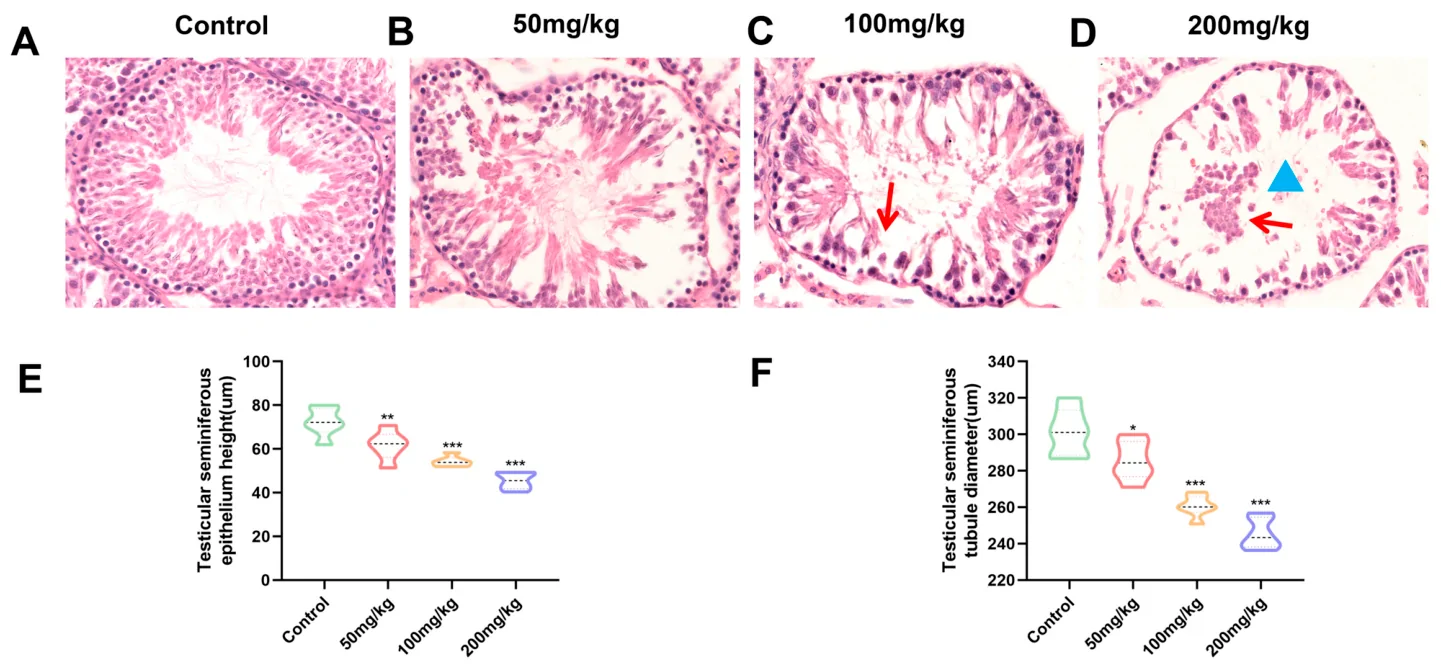
**The image (H&E staining) of SD rat testis morphological changes in arecoline-exposed (×400)**. (**A**) HE staining of testicular tissue in control group. (**B**) Testis in rats of 50 mg/kg arecoline treatment group. (**C**) Partial spermatogenic cell shed (arrows) in testicular tissue of 100 mg/kg arecoline treatment rats. (**D**) Testis in 200 mg/kg arecoline-treated rats showed that spermatogenic cell shed (arrows) and the lumina were oligozoospermic (triangle). (**E**) Effects of arecoline exposure on testicular seminiferous epithelium height. ** *p* < 0.01, *** *p* < 0.001, compared with the control group (*n* = 6). (**F**) Effects of arecoline exposure on testicular seminiferous tubule diameter. * *p* < 0.05, *** *p* < 0.001, compared with the control group (*n* = 6).

HE staining showed the morphology of rat seminiferous tubules in cross-section (Figure 1A–D). Increasing arecoline doses (50 mg/kg, 100 mg/kg, and 200 mg/kg) were associated with distinct changes in seminiferous epithelium height, seminiferous tubule diameter, and the structural integrity of spermatogenic cells. In the control group, the interstitial tissue showed no hyperplasia or edema, and spermatogenic cells were arranged in an orderly, stratified pattern. Rats treated with 50 mg/kg arecoline showed mild disorganization of the seminiferous epithelium relative to the control group. More pronounced testicular lesions were observed at 100 mg/kg and 200 mg/kg. These lesions included seminiferous tubule atrophy, detachment of the seminiferous epithelium from the basement membrane, and a significant reduction in the number of mature spermatozoa in the tubular lumen. Seminiferous epithelium height and tubule diameter also decreased in a dose-dependent manner (Figure 1E,F). These results indicate that arecoline induces dose-dependent testicular damage in rats.

### 2.3. Arecoline Exposure Reduces Epididymal Sperm Quality in Rats

**Figure 2:**
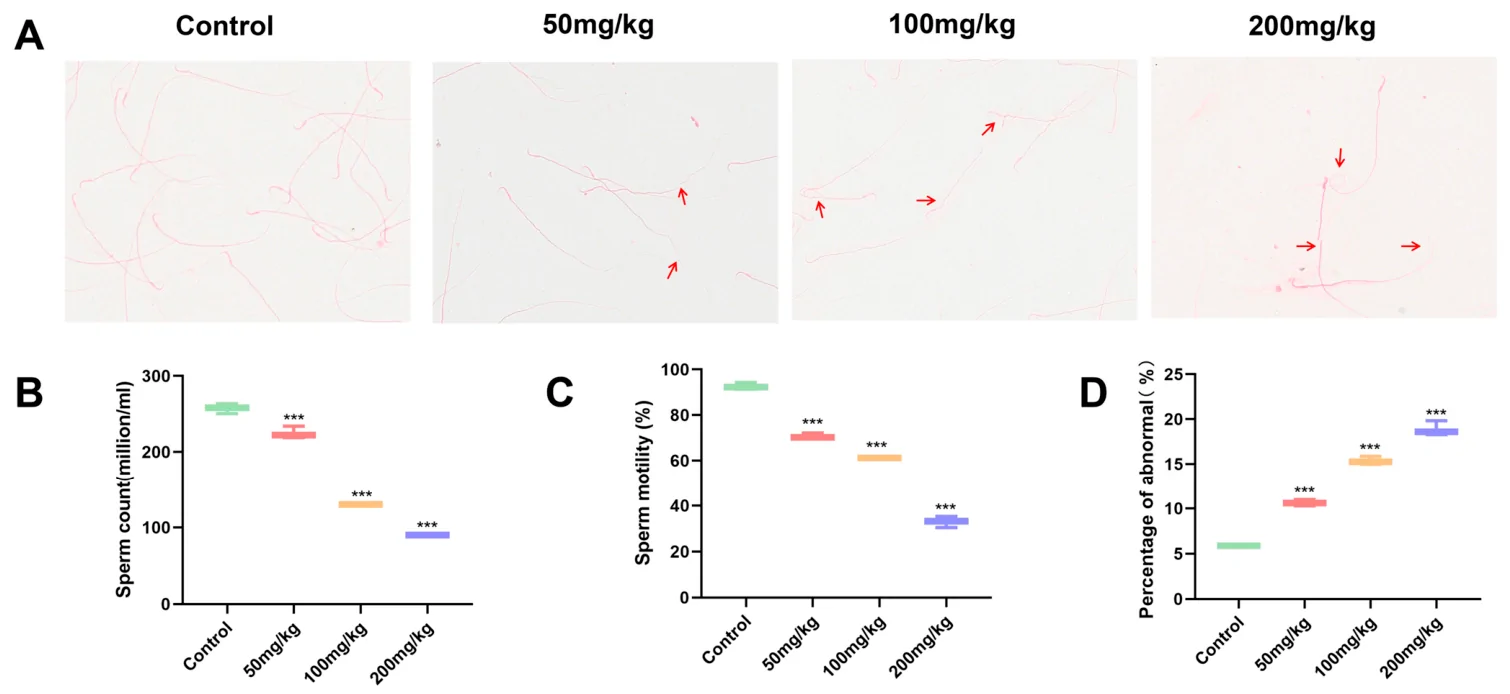
**Sperm smear of SD rat in arecoline treatment (×400).** (**A**) Sperm morphological abnormality rate was higher in the arecoline exposure group compared with the control group. The red arrows indicated abnormal spermatozoa. (**B**) Arecoline exposure decreased the sperm count in rats. *** *p* < 0.001, compared with the control group (*n* = 6). (**C**) Arecoline exposure decreased the sperm motility in rats. *** *p* < 0.001, compared with the control group (*n* = 6). (**D**) Arecoline exposure increased the percentage of abnormal sperm in rats. *** *p* < 0.001, compared with the control group (*n* = 6).

Epididymal sperm morphology was examined in arecoline-treated rats and the corresponding controls (Figure 2A). Morphologically abnormal sperm were observed in all arecoline-treated groups. Abnormalities included missing tails at 50 mg/kg; double heads, coiled tails, and missing heads at 100 mg/kg; and coiled tails and midpiece fractures at 200 mg/kg. Sperm counts and motility were significantly lower in rats treated with 50, 100, and 200 mg/kg arecoline than in the corresponding controls (Figure 2B,C). The proportion of morphologically abnormal sperm was also higher in the arecoline-treated groups and increased in a dose-dependent manner (Figure 2D).

### 2.4. Arecoline Exposure Induces Oxidative Stress in Rat Testicular Tissues

**Figure 3:**
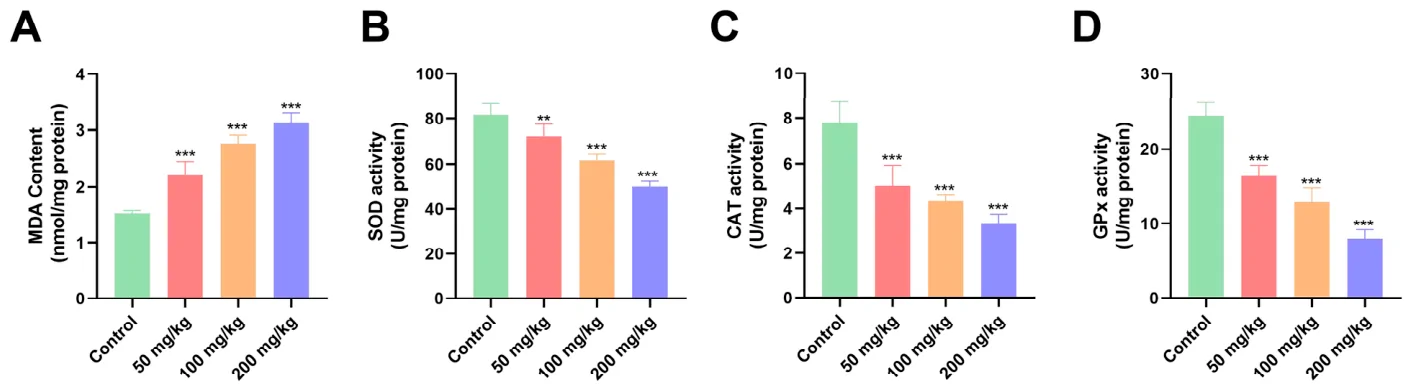
**Oxidative-stress biomarkers of testicular tissue in SD rat after arecoline treatment.** (**A**) The MDA level was higher in the arecoline exposure group compared with the control group. *** *p* < 0.001, compared with the control group (*n* = 6). (**B**) Arecoline exposure decreased the SOD activity in rat testicular tissue. ** *p* < 0.01, *** *p* < 0.001, compared with the control group (*n* = 6). (**C**) Arecoline exposure decreased the CAT activity in rat testicular tissue. *** *p* < 0.001, compared with the control group (*n* = 6). (**D**) Arecoline exposure decreased the GPx activity in rat testicular tissue. *** *p* < 0.001, compared with the control group (*n* = 6).

To assess oxidative stress in vivo, we measured MDA content and the activities of SOD, CAT, and GPx in rat testicular tissues. Compared with the control group, arecoline exposure significantly increased relative MDA levels in a dose-dependent manner (Figure 3A). The relative activities of SOD, CAT, and GPx decreased markedly with increasing arecoline dose (Figure 3B–D). These findings indicate that arecoline exposure induces oxidative stress and associated damage in testicular tissue.

### 2.5. Reduced Testicular Notch1 Expression After Arecoline Exposure Correlates with Testicular Morphometric Changes

**Figure 4:**
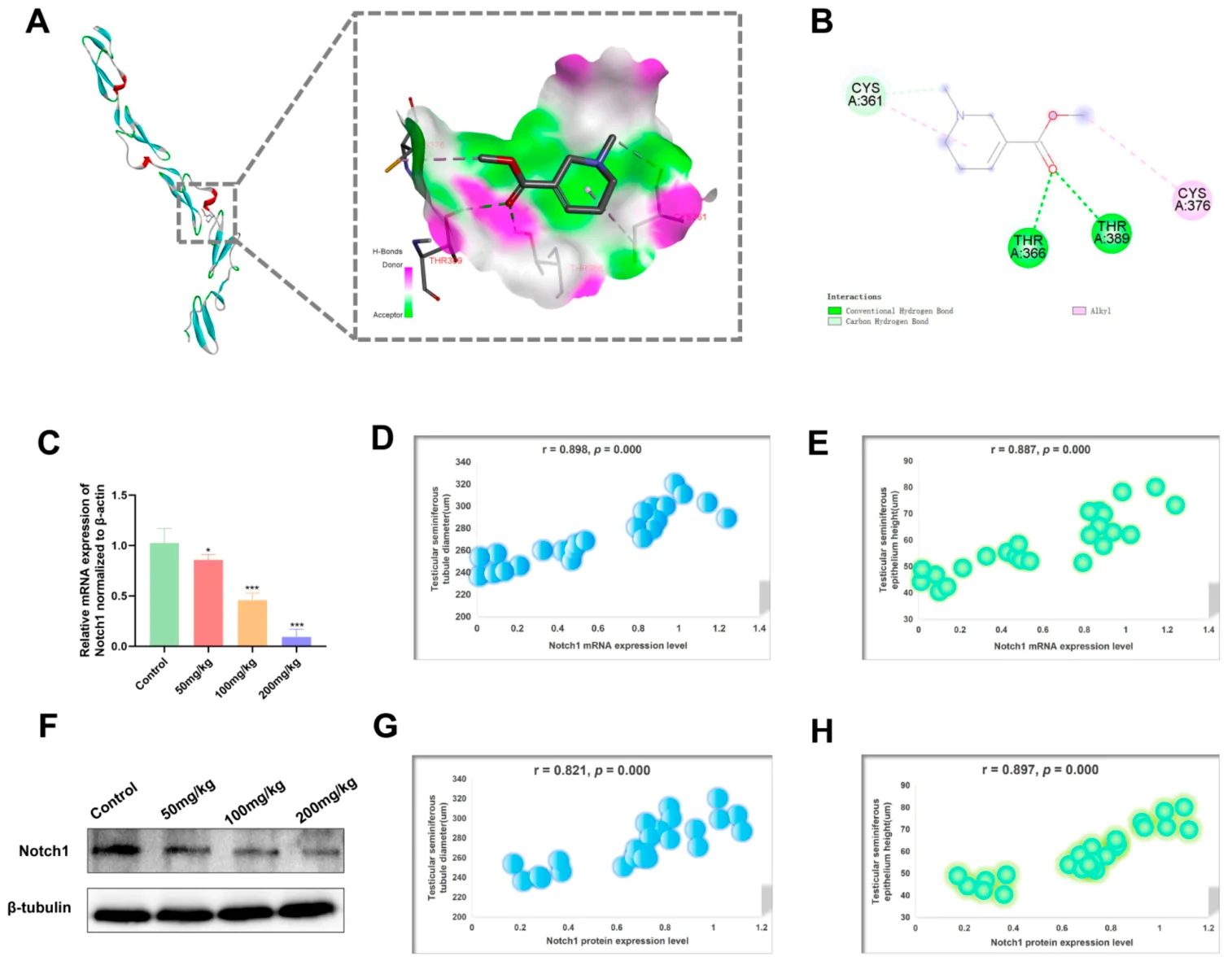
**Arecoline may interact with Notch1 and inhibit its expression; there was a correlation between the downregulation of Notch1 and the morphological parameters of testicular tissue in arecoline-exposed rats.** (**A**) Arecoline may bind the Notch1 protein. The image in the box is a 3D diagram of arecoline combined with Notch1. (**B**) The binding sites of arecoline and Notch1. (**C**) qRT-PCR showed that the mRNA expression level of Notch1 was downregulated in testicular tissues of arecoline-exposed rats compared with the control group. * *p* < 0.05, *** *p* < 0.001, compared with the control group (*n* = 6). (**D**) Results of the correlation analysis between the Notch1 mRNA expression level with testicular seminiferous tubule diameter. (**E**) Results of the correlation analysis between the Notch1 mRNA expression level with testicular seminiferous epithelium height. (**F**) Western blot demonstrated that the expression of Notch1 was downregulated in testicular tissues of arecoline-exposed rats, compared with the control group (*n* = 6). (**G**) Results of the correlation analysis between the Notch1 protein expression level with testicular seminiferous tubule diameter. (**H**) Results of the correlation analysis between the Notch1 protein expression level with testicular seminiferous epithelium height.

Spermatogenesis is the physiological process through which spermatogenic cells in the testes develop into mature sperm. This process maintains male fertility by supporting normal sperm numbers, viability, and morphology. Notch signaling may contribute to the maintenance of spermatogenesis [42]. To investigate a possible relationship between arecoline-induced spermatogenic injury and Notch signaling, we performed molecular docking. The predicted binding energy for arecoline and Notch1 was −3.5 kcal/mol, suggesting a possible interaction (Figure 4A). Analysis in PyMOL predicted hydrogen bonds between arecoline and the CYS361, THR366, and THR389 residues of Notch1, together with an alkyl interaction involving CYS376 (Figure 4B).

qRT-PCR and Western blot analyses showed that relative Notch1 mRNA and protein expression levels were significantly lower in the injured testicular tissues of arecoline-exposed rats than in the corresponding control group. These reductions in expression were particularly pronounced in rats treated with 100 and 200 mg/kg arecoline (Figure 4C,F and Figure S5A). We also performed correlation analyses between Notch1 mRNA or protein expression levels and two key testicular morphometric parameters: seminiferous tubule diameter and seminiferous epithelium height. Seminiferous tubule diameter and seminiferous epithelium height were both strongly positively correlated with Notch1 mRNA expression (r = 0.898, *p* = 0.000; r = 0.887, *p* = 0.000, respectively; Figure 4D,E). Both seminiferous tubule diameter and seminiferous epithelium height were also strongly positively correlated with Notch1 protein expression (r = 0.821, *p* = 0.000; r = 0.897, *p* = 0.000, respectively; Figure 4G,H).

### 2.6. Arecoline Alters Notch1/Jagged-1/Dll-1 Expression and Markers of Proliferation and Apoptosis in Testicular Tissues

**Figure 5:**
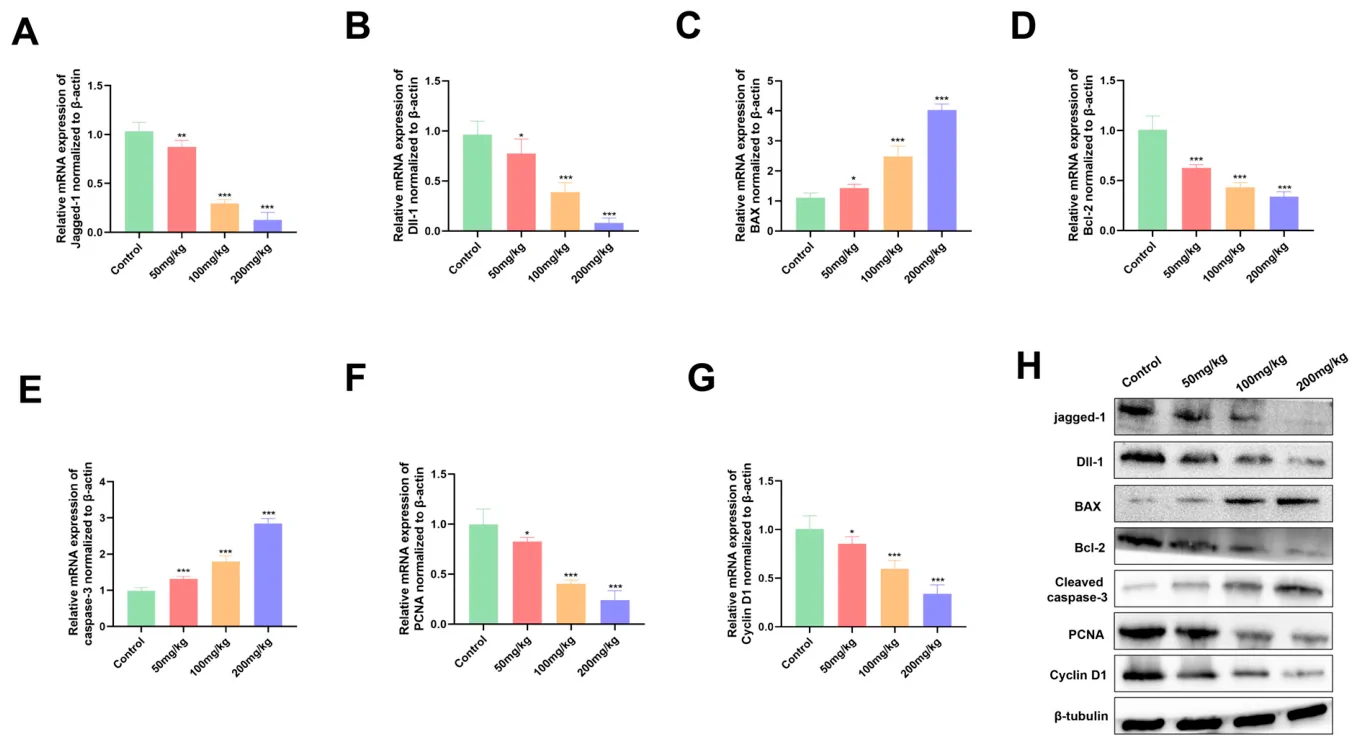
**Arecoline inhibited the proliferation of spermatogenic cells and promoted their apoptosis by regulating the Notch1/jagged-1/Dll-1 signaling pathway.** (**A**,**B**) qRT-PCR demonstrated that the expression levels of Jagged-1 and Dll-1 were gradually decreased in the testicular tissues of rats exposed to arecoline at doses ranging from 50 mg/kg to 200 mg/kg. * *p* < 0.05, ** *p* < 0.01, *** *p* < 0.001, compared with those in the control group (*n* = 6). (**C**–**E**) qRT-PCR revealed that the expression levels of BAX and caspase-3 were gradually increased, while the expression level of Bcl-2 was decreased in the testicular tissues of rats exposed to arecoline at doses ranging from 50 mg/kg to 200 mg/kg. * *p* < 0.05, *** *p* < 0.001, compared with those in the control group (*n* = 6). (**F**,**G**) qRT-PCR showed that the expression levels of PCNA and Cyclin D1 were gradually decreased in the testicular tissues of rats exposed to arecoline at doses ranging from 50 mg/kg to 200 mg/kg. * *p* < 0.05, *** *p* < 0.001, compared with those in the control group (*n* = 6). (**H**) The results of Western blot showed that the protein expression levels of Jagged-1, Dll-1, BAX, Bcl-2, cleaved caspase-3, PCNA and cyclin D1 were all consistent with the mRNA expression levels (*n* = 6).

We next assessed Notch ligand expression in the testicular tissues of rats. qRT-PCR and Western blot analyses showed a gradual decline in the mRNA and protein expression of Jagged-1 and Dll-1 in rats exposed to arecoline at doses ranging from 50 mg/kg to 200 mg/kg, compared with the corresponding control group (Figure 5A,B,H and Figure S5B,C). These findings are consistent with suppression of the Notch1/Jagged-1/Dll-1 pathway. Arecoline exposure was also associated with a significant reduction in cell proliferation and a marked increase in apoptosis relative to the control group. We used qRT-PCR and Western blotting to examine the expression of genes and proteins related to apoptosis and proliferation. The proapoptotic markers Bax and cleaved caspase-3 were upregulated, whereas the antiapoptotic protein Bcl-2 was downregulated (Figure 5C–E,H and Figure S5D–F). Concurrently, expression of the proliferation-related proteins PCNA and cyclin D1 was reduced in the testicular tissues of arecoline-exposed rats compared with the corresponding control tissues (Figure 5F–H and Figure S5H,G).

### 2.7. Arecoline Suppresses Notch Pathway Component Expression and Alters Oxidative Stress, Apoptosis, and Proliferation in GC-1 Cells

**Figure 6:**
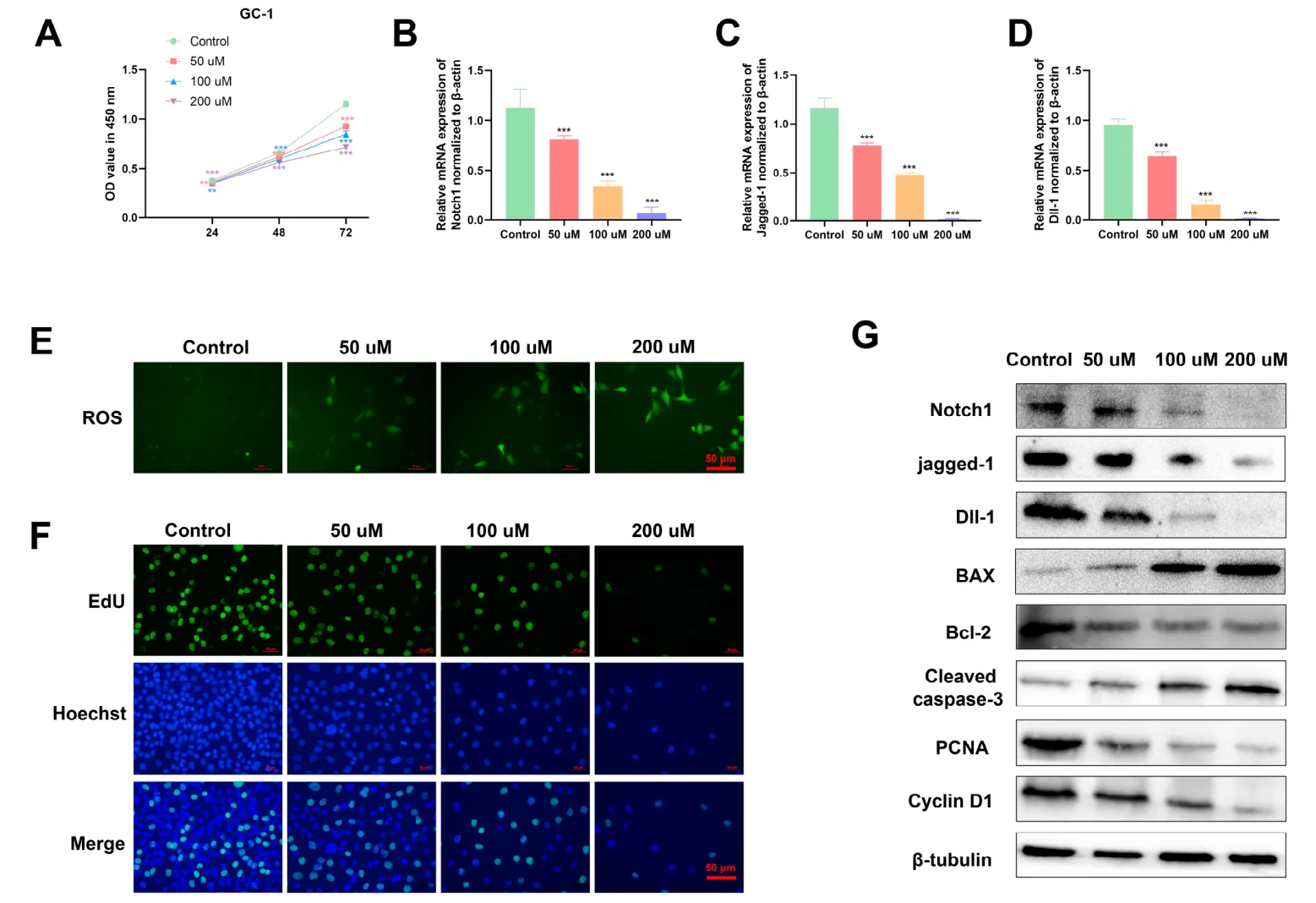
**Arecoline exposure inhibited the Notch signaling pathway in GC-1 cells, modulated Notch-mediated oxidative stress, and ultimately promoted apoptosis while inhibiting proliferation.** (**A**) CCK8 assay showed that arecoline inhibits GC-1 cell viability in a dose- and time-dependent manner. ** *p* < 0.01, *** *p* < 0.001, compared with control group. (**B**–**D**) qRT-PCR demonstrated that the mRNA expression levels of Notch-1, Jagged-1 and Dll-1 were markedly reduced in arecoline-treated cells. *** *p* < 0.001, compared with control group. (**E**) The fluorescent probe DCFH-DA revealed that the ROS generation rose with the increase in arecoline concentration. (**F**) The EdU experiment showed that 50 μM, 100 μM and 200 μM arecoline significantly inhibited the cell proliferation in GC-1 cells. (**G**) Western blot showed that the expression level of Notch1/Jagged-1/Dll-1 was decreased in arecoline-treated GC-1 cells. Bax and Cleaved caspase-3, which positively regulate apoptosis, were upregulated, whereas Bcl-2, which negatively regulates apoptosis, was downregulated, but the expression level of the proliferation-related genes PCNA and Cyclin D1 were downregulated in the arecoline-treated GC-1 cells.

To further investigate the proposed mechanism, we conducted experiments at the cellular level using GC-1 cells treated with 50 μM, 100 μM, or 200 μM arecoline. The CCK-8 assay showed that arecoline significantly reduced GC-1 cell viability in both a time-dependent and a dose-dependent manner (Figure 6A). qRT-PCR showed that the mRNA expression levels of Notch1, Jagged-1, and Dll-1 were markedly reduced in arecoline-treated cells (Figure 6B–D and Figure S5I–K). The corresponding Western blot results were consistent with these findings at the protein level (Figure 6G). Fluorescence detected using the DCFH-DA probe increased with increasing arecoline concentration, consistent with increased intracellular oxidative activity (Figure 6E and Figure S4A). The EdU assay showed reduced cell proliferation, consistent with the CCK-8 results (Figure 6F). We also used Western blot analysis to examine the expression of proliferation- and apoptosis-related proteins. Expression of Bax and cleaved caspase-3, which are associated with apoptosis, was increased, whereas expression of the antiapoptotic protein Bcl-2 was reduced. Concurrently, expression of the proliferation-related proteins PCNA and cyclin D1 was reduced in arecoline-treated GC-1 cells (Figure 6G).

### 2.8. Notch Pathway Inhibition Increases Oxidative Stress and Apoptosis and Reduces Proliferation in GC-1 Cells

**Figure 7:**
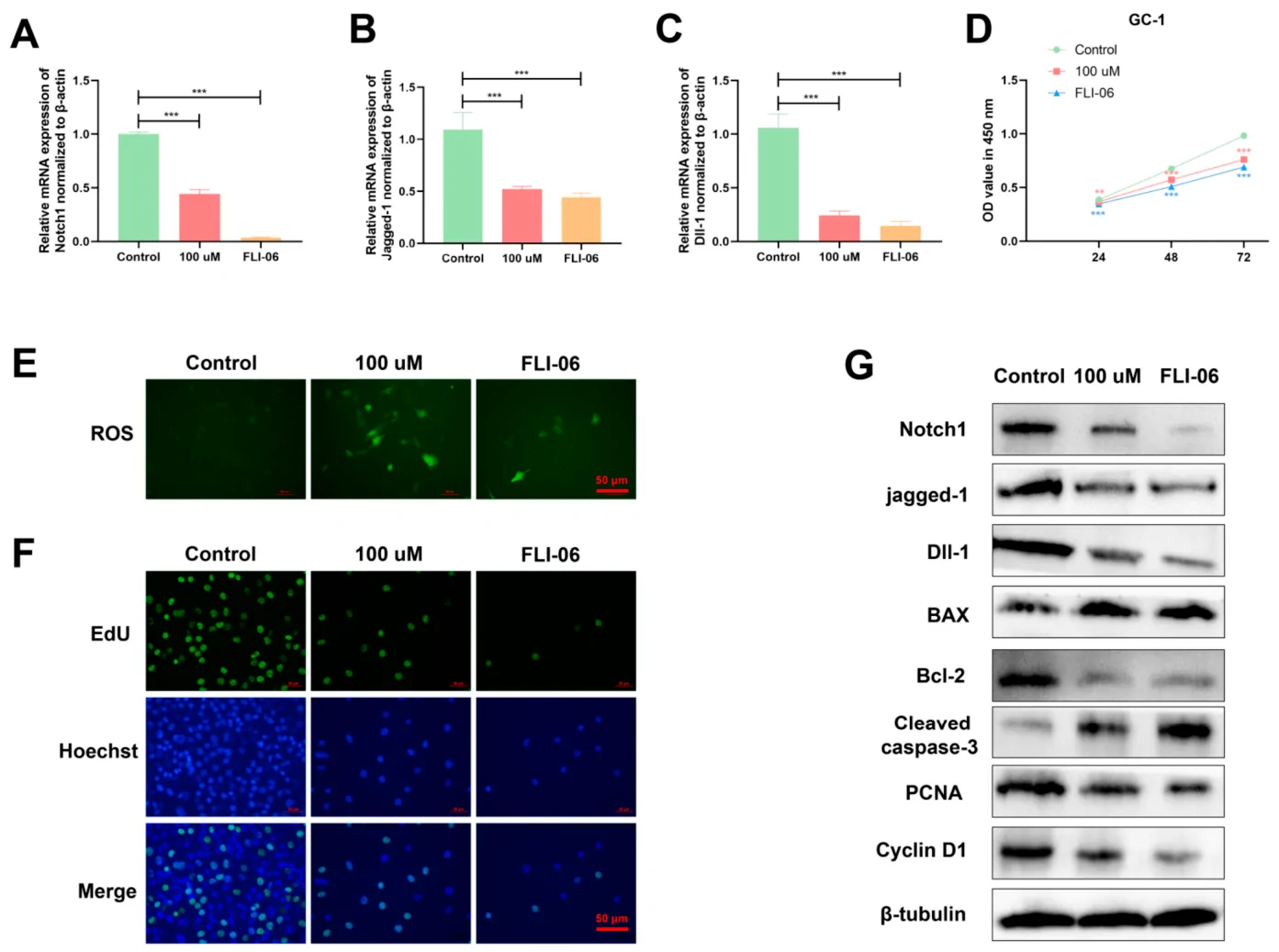
**Inhibiting the expression of Notch1 suppressed the growth of GC-1 cells.** (**A**) qRTPCR showed that Notch1 mRNA expression significantly decreased in GC-1 cells after FLI-06 treatment. *** *p* < 0.001, compared with the control group. (**B**) qRT-PCR revealed that the mRNA expression level of Jagged-1 was decreased after Notch1 inhibited. *** *p* < 0.001, compared with the control group. (**C**) qRT-PCR revealed that the mRNA expression level of Dll-1 was decreased after Notch1 inhibited. *** *p* < 0.001, compared with the control group. (**D**) CCK-8 assays showed that FLI-06 inhibited the viability of GC-1 cells. ** *p* < 0.01, *** *p* < 0.001, compared with the control group. (**E**) The fluorescent probe DCFH-DA indicated that FLI-06 significantly increased the ROS generation in GC-1 cells. (**F**) The EdU assay showed that FLI-06 inhibited the proliferation of GC-1 cells. (**G**) Western blot results confirmed that the expression level of Notch1/Jagged-1/Dll-1 was decreased after FLI-06 treatment in GC-1 cells. Bax and Cleaved caspase-3, which positively regulate apoptosis, were upregulated, whereas that of Bcl-2, which negatively regulates apoptosis, was downregulated; however, the expression level of the proliferation-related genes PCNA and cyclin D1 were downregulated in the FLI-06-treated GC-1 cells.

To further investigate the functional role of Notch1 in GC-1 cells, we used FLI-06, an inhibitor of Notch signaling. Treatment with FLI-06 significantly reduced Notch1 expression at both the mRNA and protein levels (Figure 7A,G). Consistent with this reduction, expression of the Notch ligands Jagged-1 and Dll-1 was also reduced at both the mRNA and protein levels following FLI-06 treatment (Figure 7B,C,G). We then conducted functional assays to assess the cellular effects associated with this intervention. The CCK-8 and EdU assays showed that FLI-06 significantly reduced the viability and proliferative capacity of GC-1 cells, respectively (Figure 7D,F). Reduced Notch1 expression was accompanied by significantly increased ROS-associated fluorescence in GC-1 cells (Figure 7E and Figure S4B). Western blot analysis showed increased expression of the proapoptotic markers Bax and cleaved caspase-3 and reduced expression of the antiapoptotic protein Bcl-2. Expression of the proliferation-related proteins PCNA and cyclin D1 was also reduced in FLI-06-treated GC-1 cells (Figure 7G). The effects of FLI-06 on oxidative activity, apoptosis-related markers, and proliferation resembled those observed in arecoline-treated cells. These findings provide further support for the involvement of reduced Notch signaling in arecoline-induced GC-1 cell injury, although pharmacological inhibition alone cannot establish pathway specificity.

### 2.9. Enhanced Notch Signaling Is Associated with Reduced Oxidative Stress and Apoptosis and Improved Proliferation in Arecoline-Treated GC-1 Cells

**Figure 8:**
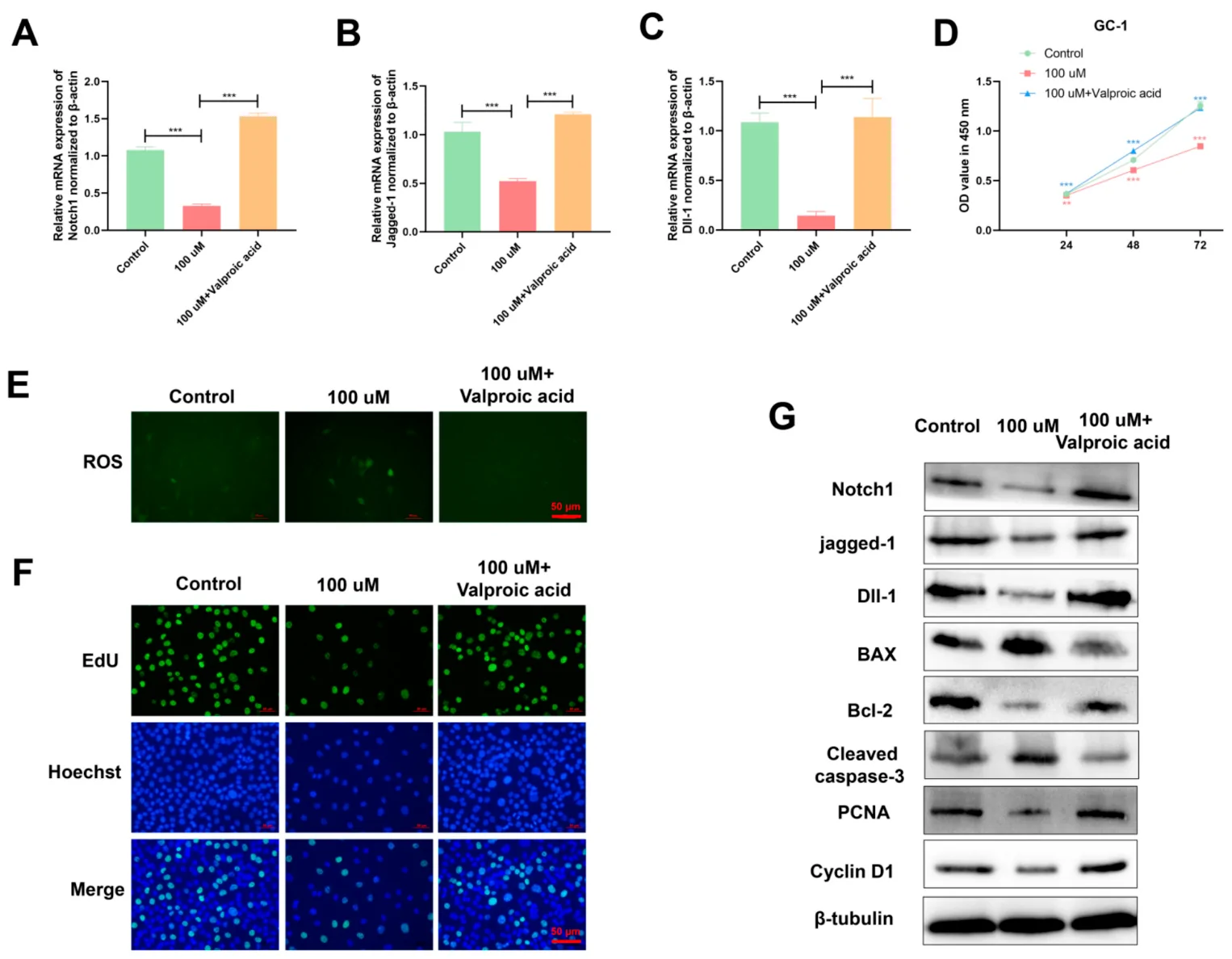
**Activation of the Notch signaling pathway alleviated the oxidative stress in GC-1 cells treated with arecoline, thereby reducing the apoptosis of GC-1 cells and promoting their proliferation.** (**A**) qRT-PCR revealed that Notch1 mRNA expression significantly increased after valproic acid treatment in GC-1 cells. *** *p* < 0.001, compared with the arecoline-treated group. (**B**) qRT-PCR revealed that Jagged-1 mRNA expression significantly increased after valproic acid treatment in GC-1 cells. *** *p* < 0.001, compared with the arecoline-treated group. (**C**) qRT-PCR revealed that Dll-1 mRNA expression significantly increased after valproic acid treatment in GC-1 cells. *** *p* < 0.001, compared with the arecoline-treated group. (**D**) CCK8 assays showed that the viability of GC-1 cells increased after treatment with valproic acid. ** *p* < 0.01, *** *p* < 0.001, compared with the arecoline-treated group. (**E**) The fluorescent probe DCFH-DA demonstrated that ROS production was significantly reduced in GC-1 cells treated with valproic acid, compared with the arecoline-treated group. (**F**) The EdU experiment showed that proliferation ability was restored in GC-1 cells treated with valproic acid, compared with the arecoline-treated group. (**G**) Western blot analysis confirmed that valproic acid treatment upregulated the expression levels of Notch1, Jagged-1, and Dll-1 in GC-1 cells compared with the arecoline-treated group. Concomitantly, the expression of the pro-apoptotic proteins Bax and Cleaved caspase-3 was downregulated, while that of the anti-apoptotic protein Bcl-2 was upregulated. Additionally, in Notch1-upregulated GC-1 cells, the expression of the proliferation-related proteins PCNA and Cyclin D1 was increased.

To determine whether Notch1 contributes to arecoline-induced spermatogenic injury, we treated GC-1 cells with valproic acid (VPA), which was used to enhance Notch1 signaling. VPA treatment significantly increased Notch1 mRNA expression (Figure 8A). Consistent with this increase, the mRNA expression levels of its ligands, Jagged-1 and Dll-1, were also significantly increased (Figure 8B,C). Western blot analysis confirmed corresponding increases at the protein level (Figure 8G). We next assessed the effects of this intervention on the biological responses of GC-1 cells. The CCK-8 assay showed that cell viability improved following VPA treatment compared with that of cells exposed to arecoline alone (Figure 8D). Increased Notch1 expression was also accompanied by an apparent reduction in ROS-associated fluorescence (Figure 8E and Figure S4C) and increased proliferation relative to arecoline treatment alone (Figure 8F). Western blot analysis showed reduced expression of Bax and cleaved caspase-3, which are associated with apoptosis, and increased expression of the antiapoptotic protein Bcl-2. Concurrently, expression of the proliferation-related proteins PCNA and cyclin D1 was increased in GC-1 cells with elevated Notch1 expression (Figure 8G). Importantly, VPA alone exerted no significant effects on cell proliferation, ROS accumulation, or the expression of apoptosis-associated proteins in GC-1 cells (Figure S3), indicating that the rescue effect of VPA was specific to arecoline-induced cellular damage, rather than a direct independent effect of VPA.

### 2.10. siRNA-Mediated Notch1 Silencing Reproduces Arecoline-Induced Cellular Changes in GC-1 Cells

**Figure 9:**
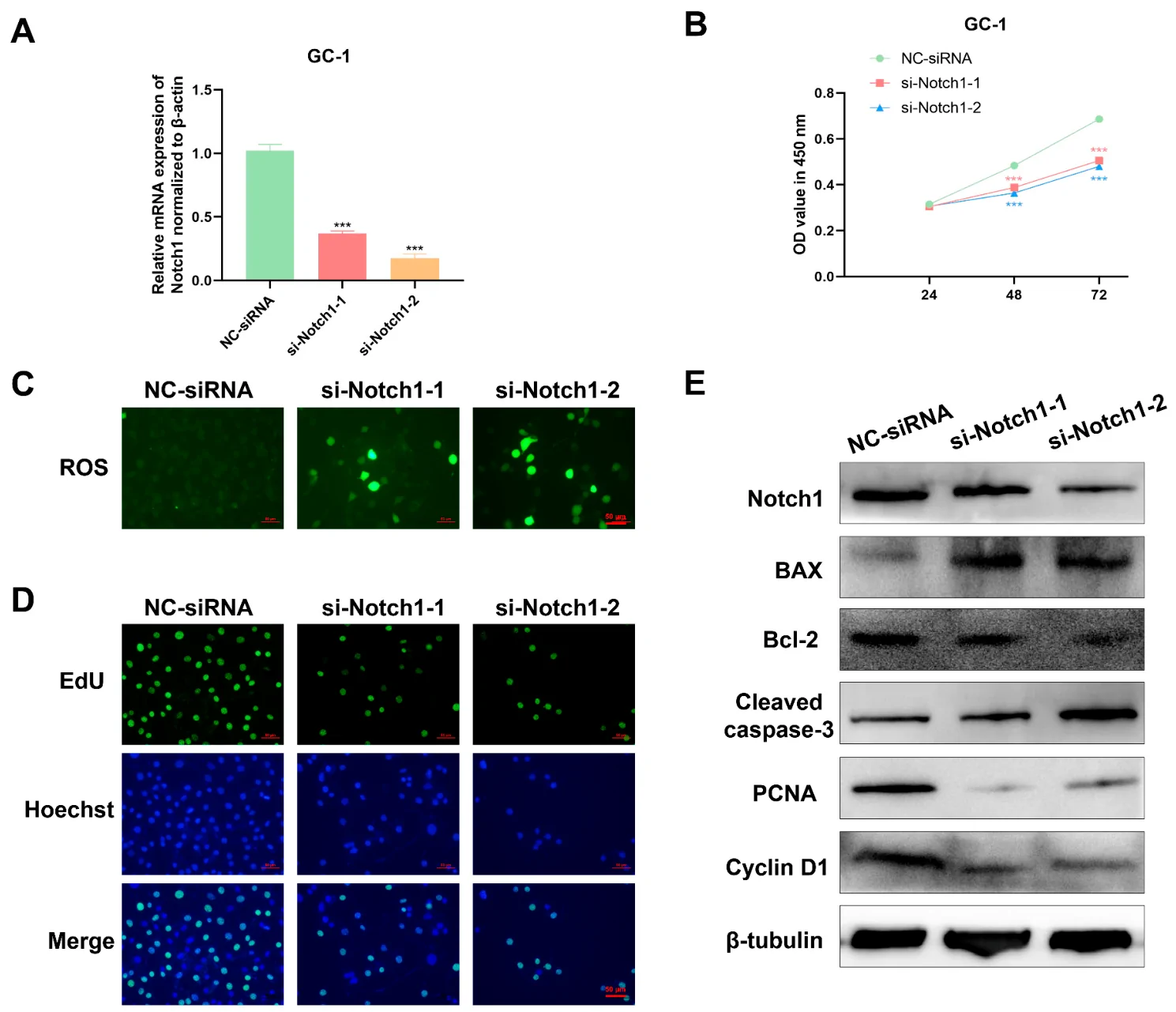
**si-RNA-mediated Notch1 knockdown reproduces arecoline-induced damage phenotypes in GC-1 cells.** (**A**) qRT-PCR revealed that Notch1 mRNA expression significantly decreased after si-Notch1 transfection in GC-1 cells. *** *p* < 0.001, compared with the si-NC group. (**B**) CCK-8 assays showed that the viability of GC-1 cells decreased after si-Notch1 transfection. *** *p* < 0.001, compared with the si-NC group. (**C**) The fluorescent probe DCFH-DA demonstrated that ROS production was significantly elevated in GC-1 cells after si-Notch1 transfection, compared with the si-NC group. (**D**): The EdU experiment showed that proliferation ability was impaired in GC-1 cells after si-Notch1 transfection, compared with the si-NC group. (**E**) Western blot analysis confirmed that si-Notch1 transfection downregulated the expression levels of Notch1 in GC-1 cells compared with the si-NC group. Concomitantly, the expression of the pro-apoptotic proteins Bax and Cleaved caspase-3 was upregulated, while that of the anti-apoptotic protein Bcl-2 was downregulated. Additionally, in Notch1-silenced GC-1 cells, the expression of the proliferation-related proteins PCNA and Cyclin D1 was decreased.

To further investigate the causal contribution of Notch1 to GC-1 cell injury and complement the pharmacological intervention experiments, we performed siRNA-mediated Notch1 knockdown without arecoline treatment. Transfection with si-Notch1 markedly reduced Notch1 mRNA and protein expression compared with the negative-control siRNA (si-NC) group (Figure 9A,E). Notch1 knockdown alone significantly reduced cell viability (Figure 9B), increased intracellular ROS-associated fluorescence (Figure 9C and Figure S4D), and impaired proliferation, as assessed by EdU staining (Figure 9D). Western blot analysis showed that Notch1 silencing increased the expression of pro-apoptotic Bax and cleaved caspase-3 and reduced the expression of anti-apoptotic Bcl-2. The proliferation-related proteins PCNA and cyclin D1 were also significantly downregulated following Notch1 knockdown (Figure 9E).

These changes closely resembled those observed after arecoline treatment, supporting a functional role for Notch1 downregulation in arecoline-induced spermatogonial injury.

**Figure 10:**
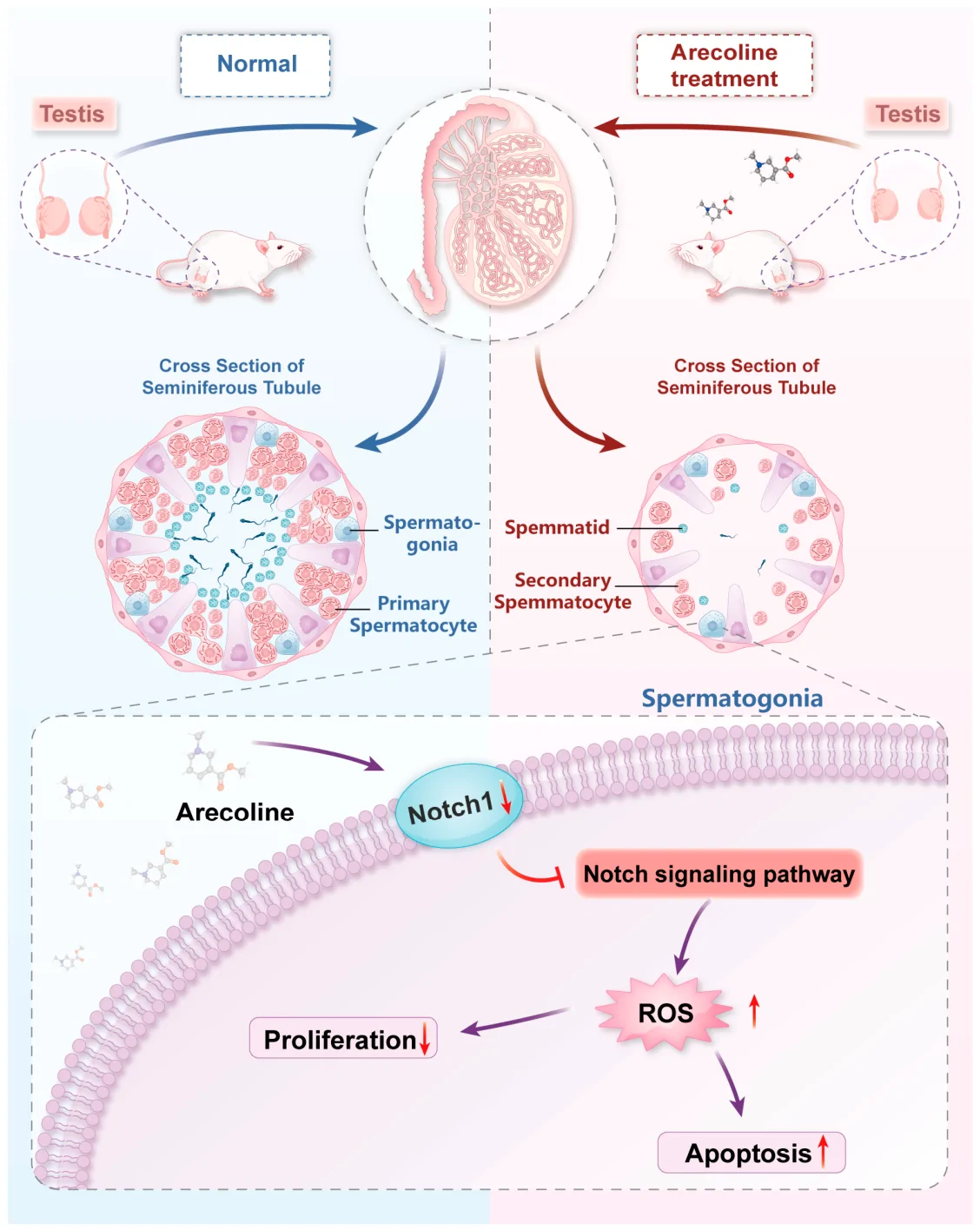
**Diagram of the proposed mechanism by which arecoline induces testicular and spermatogenic injury in rats.**

Collectively, these findings demonstrate that arecoline exposure triggers testicular and spermatogenic injury in rats. The underlying mechanism is associated with oxidative stress mediated by the Notch signaling pathway. Arecoline may bind to Notch1 and reduce its expression, leading to inhibition of Notch signaling, excessive accumulation of ROS, increased apoptosis and impaired proliferation of spermatogenic cells (Figure 10).

In testicular tissues, arecoline exposure could induce spermatogenic injury through Notch signaling-mediated oxidative stress. Specifically, arecoline may interact with Notch1 and downregulate its expression. Reduced Notch1 expression suppresses the Notch signaling pathway, elevates ROS production, and thereby induces apoptosis and inhibits proliferation in spermatogenic cells.

## 3. Discussion

Male infertility is a major reproductive health concern worldwide, and male factors contribute to approximately 50% of infertility cases [1]. Chemical toxicants are an important cause of male infertility [43,44]. Areca nut is the fourth most widely consumed psychoactive substance worldwide [10]. Arecoline, a tetrahydropyridine alkaloid in areca nut, has several reported pharmacological activities, including antiparasitic, digestive, analgesic, anti-inflammatory, and antiallergic effects. It is also a toxic constituent of areca nut [45]. Previous studies have investigated its toxic effects and mechanisms in multiple organs [46,47,48]. Preliminary reproductive studies have reported adverse effects on reproductive function [49,50], but the underlying molecular mechanisms remain unclear. We therefore evaluated the dose-dependent effects of arecoline and investigated mechanisms of male reproductive toxicity. Male rats exposed to 50 mg/kg, 100 mg/kg, or 200 mg/kg arecoline exhibited several signs of reproductive toxicity. Notch1 expression was markedly reduced in their testicular tissues, and lower expression was closely associated with poorer testicular morphometric measures. We also examined changes in Notch signaling and oxidative injury after arecoline exposure. The results support the possibility that altered Notch signaling contributes to increased ROS-associated oxidative activity and disrupted spermatogonial proliferation and apoptosis. These changes were accompanied by reduced sperm counts and motility and a higher proportion of abnormal sperm. The findings suggest a potential molecular pathway underlying the reproductive toxicity of arecoline.

Spermatogenesis sustains male fertility through three sequential stages: spermatogonial proliferation and differentiation, the meiotic division of spermatocytes, and the differentiation of spermatids through spermiogenesis [51]. Evidence supports a regulatory role for Notch signaling in this process [42,52,53]. Parekh et al. [38] reported that undifferentiated spermatogonial cells regulate Cyp26b1 expression through Notch signaling, thereby contributing to germ cell differentiation. Murta et al. showed that Notch pathway components and effectors are expressed at the transcriptional level in testicular germ cells, Sertoli cells, and stromal cells. Their cell-type-specific and time-dependent expression patterns suggest roles in testicular development, including prepubertal spermatogonial quiescence, the initiation of spermatogenesis, and regulation of the spermatogenic cycle [54]. Abnormal Notch signaling has also been associated with male infertility [39].

Molecular docking predicted a possible interaction between arecoline and Notch1, with a binding energy of −3.5 kcal/mol and hydrogen bonds involving residues including CYS361 and THR366. In vivo, arecoline exposure significantly reduced the mRNA and protein expression of Notch1 and its ligands, Jagged-1 and Dll-1, in rat testicular tissues. Notch1 expression was strongly positively correlated with seminiferous tubule diameter and seminiferous epithelium height. These findings associate arecoline exposure with reduced Notch pathway component expression and reproductive impairment. They support further investigation of Notch1 as a potential molecular target, while direct binding and the relationship between binding and reduced expression remain to be established.

Arecoline can induce several forms of cellular injury, including morphological disruption, reduced survival, abnormal physiological function, and apoptosis. Proposed mechanisms include increased ROS levels, altered autophagy, epigenetic dysregulation, and immune dysfunction [55,56]. Oxidative stress is a major mechanism implicated in areca nut toxicity. In the nervous system, for example, arecoline-induced neurotoxicity is associated with enhanced oxidative stress and impaired antioxidant defenses [57]. In the liver, toxicity involves oxidative stress, hepatocyte ultrastructural damage, and elevated liver enzyme levels [58]. In oral epithelial cells, arecoline-induced oxidative stress is accompanied by inflammatory mediator production and altered keratin expression [59]. Spermatogonial populations include stem cells and proliferating progenitors. Their repeated cell-cycle entry and DNA replication may increase their susceptibility to environmental toxicants. Damage to these populations can deplete the cells available to generate spermatocytes, thereby disrupting spermatogenesis. Spermatogonial cells may therefore be an important target of arecoline-induced reproductive injury. We used the GC-1 spermatogonial cell line to investigate whether oxidative stress contributes to this injury in vitro. Treatment with 50, 100, and 200 μM arecoline increased ROS-associated fluorescence and was accompanied by reduced proliferation and increased apoptosis. These cellular changes provide a possible explanation for impaired spermatogenesis in vivo. Testicular biochemical measurements also showed increased MDA levels and reduced SOD, CAT, and GPx activities after arecoline exposure, providing in vivo evidence of oxidative stress. These observations prompted us to investigate whether altered Notch signaling contributes to the oxidative response in GC-1 cells.

We next examined whether Notch signaling contributes to arecoline-induced oxidative stress. Pharmacological inhibition of the pathway increased oxidative stress-associated fluorescence and apoptosis and reduced GC-1 cell proliferation, resembling the effects of arecoline exposure. Conversely, pharmacological enhancement of Notch signaling in arecoline-treated cells reduced oxidative stress-associated fluorescence and apoptosis and improved proliferation. The siRNA experiments provided complementary evidence, as Notch1 knockdown reproduced the injury-associated cellular changes without arecoline exposure. These results support a role for Notch1 in maintaining the oxidative balance of spermatogonial cells under the tested conditions.

Several limitations of the present study should be acknowledged. Intracellular oxidative activity in GC-1 cells was measured with the DCFH-DA fluorescent probe, which generates a nonspecific oxidation-dependent signal and cannot distinguish individual ROS subtypes. Although we detected classic oxidative-stress biomarkers (MDA, SOD, CAT, GPx) in rat testicular tissue, mitochondrial morphology, function and total ATP levels were not evaluated. Given that mitochondria constitute a major source of cellular ROS, further work on mitochondrial homeostasis and ATP metabolism will help clarify arecoline-triggered spermatogenic injury. Molecular docking only predicts a potential arecoline-Notch1 interaction and cannot prove direct physical binding. Biochemical assays such as surface plasmon resonance (SPR) or suitably designed co-immunoprecipitation (Co-IP) are required to validate this small-molecule–protein binding event. In addition, our in vivo model adopted intraperitoneal injection in SD rats, and in vitro experiments relied on the immortalized GC-1 spermatogonial cell line. Species disparities, exposure route and the non-primary cell nature restrict direct extrapolation of these results to human areca-nut-chewing populations; future studies using primary spermatogonia and oral-exposure animal models are warranted. Finally, while our data demonstrate that Notch1 mediates arecoline-induced spermatogonial damage by regulating ROS accumulation, the specific downstream target genes of Notch1 signaling remain uncharacterized, which will be addressed in our follow-up work.

In conclusion, our findings support a role for altered Notch signaling and oxidative stress in arecoline-induced spermatogenic injury. Specifically, arecoline may interact with the Notch1 protein, resulting in the downregulation of Notch1 expression and inhibition of its signaling pathway. This subsequently induces oxidative stress both in spermatogonial cells and rat testicular tissue, suppresses spermatogonial proliferation, and triggers apoptosis. This effect is one of the potential mechanisms underlying the testicular damage that occurs in rats exposed to arecoline. Pharmacological interventions and Notch1 knockdown provided complementary evidence for the involvement of this pathway. Our research fills a key gap in our understanding of the reproductive toxicity of arecoline.

## 4. Materials and Methods

### 4.1. Arecoline Preparation and Animals Experiments

Arecoline standard (HPLC ≥ 98%, CAS: 63-75-2) were obtained from Shanghai Yuanye Biotech Co. Ltd. (Shanghai, China). Male SD (Sprague–Dawley) rats were obtained from the Laboratory Animal Center of Xi’an Jiaotong University. The rats (aged 4 weeks old, about 130–150 g) were housed under standard laboratory conditions (temperature 22 ± 2 °C, 12 h day/night). Animals had ad libitum access to food and water. After one week of acclimatization, rats were stratified and randomly allocated into four groups according to baseline body weight. Briefly, rats were divided into weight strata before allocation, with randomization within each stratum to achieve balanced baseline body weight across groups. Statistical analysis of baseline weight data after grouping confirmed no significant intergroup differences (as shown in the data from Day 0 in Figure S2).

Sample size (6 rats per group, 4 groups, total *n* = 24) was selected using Mead’s resource equation [60]: (E = *n* − k = 24 − 4 = 20), falling within the recommended range of 10–20. This sample size is consistent with common practice in male reproductive toxicology studies [61,62,63,64] using SD rats and follows the 3R principle to minimize animal use. A sensitivity analysis was conducted to estimate the minimum detectable effect size of this experimental design.

Arecoline was administered via intraperitoneal injection at doses of 50, 100, and 200 mg/kg body weight once daily for 30 consecutive days. The corresponding control group received sterile saline. Based on published human exposure data [65], the high-exposure arecoline intake of heavy areca-nut chewers was scaled to approximately 27 mg/kg/day for rats using body-surface-area conversion. Preliminary animal studies showed that this theoretical human-equivalent dose only elicited weak and unstable spermatogenic changes after 30-day administration. Hence, 50 mg/kg/day (1.8-fold human-equivalent exposure) was set as the minimum dose, which was biologically relevant to heavy areca-nut consumers and could induce measurable early testicular damage. The 100 mg/kg/day dose was adopted according to previous toxicological studies reporting obvious testicular oxidative stress and spermatogenic impairment after 30-day exposure to areca-nut-derived alkaloids [29]. The 200 mg/kg/day was the upper limit of our dose gradient to construct a complete dose–response curve for arecoline-triggered testicular injury. This dose was 7.4-fold of the human-equivalent level; higher doses would go beyond human-relevant exposure scenarios and would only exacerbate tissue damage without supplying extra mechanistic data, while bringing unnecessary animal suffering. No death occurred in the 200 mg/kg/day group throughout the 30-day treatment.

### 4.2. Samples Preparation

At the end of the experiment, rats received intraperitoneal injection of pentobarbital sodium at 50 mg/kg to achieve deep surgical anesthesia. Adequate anesthetic depth was verified by the loss of both pedal withdrawal and corneal reflexes. Subsequently, cervical dislocation was applied as a secondary physical euthanasia procedure to ensure irreversible death. Both testes and cauda epididymis were then dissected. Sperm count, motility and morphology were calculated. In addition, the left testis was applied for histopathological examination, which was fixed in the fresh Formalin solution; the right testis was applied for detection of molecular and biochemical indicators, which was stored at −80 °C.

### 4.3. Histopathological Analysis and Testicular Morphometry

The testis was excised and fixed with a fresh formalin solution overnight at room temperature. Tissues were then embedded in paraffin and sectioned into 4 μm thick slices, followed by haematoxylin and eosin (H&E) staining. Finally, the testicular tissues’ histopathological morphology was observed under a light microscope (BX51, Olympus, Tokyo, Japan).

According to our previous methods [62], these indicators, including testicular seminiferous epithelium height and seminiferous tubule diameter, were quantified. For testicular morphometric analysis, five non-overlapping random visual fields were captured from each testicular cross-section, avoiding tissue marginal regions. For each field, 8–10 circular or nearly circular seminiferous tubules were selected, yielding no fewer than 40 measured tubules per rat. The seminiferous epithelium height and seminiferous tubule diameter were measured. All measurements were conducted by an investigator blinded to experimental group information. Multiple tubule measurements from a single rat were averaged to obtain one representative value per animal. Statistical analyses were performed using rat-level averaged data, and each rat was treated as one biological replicate (*n* = 6 per group).

### 4.4. Collection of Epididymal Sperm

Epididymal sperm were harvested by making 1–2 longitudinal incisions with ophthalmic scissors and followed by perfusion with pre-warmed 0.9% normal saline at 37 °C. The epididymal perfusate was centrifuged at 225× *g* for 10 min. The pellet was resuspended in 1.0 mL of normal saline. An aliquot of the sperm suspension was subjected to assessments of sperm count, motility, and morphology.

### 4.5. Epididymal Sperm Count and Sperm Motility

Sperm count and motility assessment for epididymal samples were conducted according to the protocols described by Joshi et al. [66] and the WHO manual for semen analysis [67]. Briefly, sperm count and motility were evaluated microscopically within a counting chamber. The sperm count was calculated in 10 fields of the counting chamber and expressed as 10^6^ per sperm dilution. Sperm motility was determined by counting both motile and non-motile sperms in at least 10 separate and randomly selected fields and percent motility was calculated as (number of motile sperm/total number of sperm counted, i.e., motile plus non-motile sperm) × 100%. These results were expressed as percent motility. The count was repeated three times for each sample to minimize error. No human semen reference limits were applied to the rat data; all sperm parameters were compared with those of the concurrent control rats. All evaluations were performed by an evaluator blinded to the treatment groups.

### 4.6. Epididymal Sperm Morphology

The sperm suspension was filtered through a 40 μm cell filter, and then 20 μL filtered suspension smear was collected. For each animal, three smears were prepared. After the sections were dried, they were fixed with ice methanol (Solarbio, Beijing, China) for 5 min and air dried. Finally, the sections were stained with 2% eosin (Solarbio, Beijing, China) for 1 h and rinsed with water for 5 min. Finally, 200 spermatozoa per smear (600 spermatozoa per rat) were counted at 400× magnification under light microscopy. A spermatozoon was recorded as abnormal if it showed head defects (amorphous, banana-shaped, hookless or detached) or tail defects (coiled, folded, bent, double or broken), following the criteria established for rodent sperm-abnormality assays [68,69]. Spermatozoa overlapping neighboring cells that could not be classified unambiguously were excluded. The percentage of abnormal sperm was calculated. The evaluator was blinded to the treatment groups.

### 4.7. Cell Culture and Treatment

The mouse spermatogonial cell line GC-1 spg (ATCC, Rockville, MD, USA) was cultured in Dulbecco’s Modified Eagle’s Medium (DMEM) (Hyclone, South Logan, UT, USA) supplemented with 10% fetal bovine serum (Gibco, Thermo Fisher Scientific, Waltham, MA, USA) and antibiotics (100 IU/mL penicillin, 100 µg/mL streptomycin, Hyclone). A broad arecoline concentration panel (0, 12.5, 25, 50, 100, 200, 400 μM) was screened in our preliminary experiments. Concentrations of 12.5 and 25 μM did not induce obvious detectable cellular toxic phenotypes, whereas 400 μM caused severe non-specific cell injury and massive necrosis that would interfere with mechanistic analysis (Figure S1). Consequently, 50, 100 and 200 μM were selected as the working arecoline doses for formal experiments.

### 4.8. Molecular Docking

The SDF-format file for arecoline was obtained from the PubChem database, whereas the three-dimensional structure of the Notch1 protein was retrieved from the PDB database (PDB ID: 5UK5). The target protein was preprocessed, including water molecules and extrinsic small-molecule ligand removal. using PyMOL software (version 2.1.0). Next, hydrogen addition and charge assignment were performed on the optimized protein via AutoDock Tools (version 1.5.6), and the processed protein was exported in “.pdbqt” format.

Molecular docking was performed with Vina 2.0 integrated in PyRx (version 0.8) software, with Notch1 serving as the receptor and arecoline as the ligand. The binding energy was calculated, and output files were generated. The affinity value (kcal/mol) represents the binding capacity between the ligand and the receptor; specifically, a lower affinity value indicates a more stable binding interaction between the ligand and the receptor. Finally, docking outputs were visualized using Discovery Studio 2020 Client (available at https://discover.3ds.com/discovery-studio-visualizer-download (accessed on 29 June 2025).

### 4.9. Cell Viability Assay

Four groups of GC-1 spg cells were set up in this experiment: control group, low-dose arecoline group, medium-dose arecoline group, and high-dose arecoline group. GC-1 spg cells were seeded in 96-well plates at a density of 5000 cells/well in 100 μL DMEM. Once cells had adhered, they were exposed to varying arecoline concentrations and cultured for 24, 48, and 72 h separately. Upon completion of incubation, the cells were treated with CCK-8 reagent (Meilunbio, DaLian, China) and incubated for 3.5 h. Then, an enzyme standard device (Thermo Fisher Scientific, Waltham, MA, USA) was used to measure the optical density (OD) of the cells at 450 nm. All experiments were performed in triplicate.

### 4.10. EdU Cell Proliferation Assay

Cell proliferation was evaluated using the EdU Apollo567 Imaging Kit (RiboBio, Guangzhou, China). GC-1 cells were seeded in 24-well plates at a density of 5 × 10^4^ cells per well. After cell attachment, cells were exposed to varying concentrations of arecoline, and incubated for an additional 24 h. Subsequently, cells were incubated with 50 μM EdU for 2 h (37 °C, 5% CO_2_). After fixation in 4% paraformaldehyde for 30 min and permeabilization with 0.5% Triton X-100 in PBS for 10 min, EdU incorporation was visualized by incubation with Apollo567 reaction cocktail for 30 min. Nuclei were counterstained with Hoechst 33342 (5 μg/mL, 15 min). Fluorescence images were captured with an inverted fluorescence microscope (Olympus). All experiments were performed in triplicate.

### 4.11. Reactive Oxygen Species (ROS) Assay

Intracellular ROS levels were evaluated using a commercial ROS assay kit (Beyotime, Shanghai, China). The cell-permeable probe DCFH-DA was applied to monitor intracellular ROS generation. The GC-1 cells were seeded in 96-well plates and cultured until the cell confluence reached approximately 70–80%, cells were then exposed to varying arecoline concentrations and incubated for an additional 48 h. Afterwards, the culture medium was replaced with DMEM supplemented with 25 μM DCFH-DA. Following a 30 min incubation in the dark, DCFH-DA-derived fluorescence was visualized with a fluorescence microscope (BX53, Olympus, Tokyo, Japan) at an excitation wavelength of 488 nm.

### 4.12. RNA Extraction and Quantitative Real-Time Polymerase Chain Reaction (qRT-PCR) Analysis

Total RNA was isolated from the testes samples and GC-1 cells using TRIzol reagent (Invitrogen, Carlsbad, CA, USA). Isolated RNA was reverse-transcribed into cDNA with a Takara Reverse Transcription Kit (Takara, Kusatsu, Japan). Reaction mixtures were prepared using SYBR Green qPCR Master Mix (Cwbio, Beijing, China). The reactions were implemented on Bio-RadCFX Connect real-time system (Bio-Rad, Hercules, CA, USA). Relative target mRNA expression was normalized to β-actin and Hprt1. Gene expression data were calculated via the 2^−ΔΔCT^ method. Detailed primer sequences for qRT-PCR are provided in Table 2.

In this study, β-actin and Hprt1 were selected as candidate reference genes for qPCR normalization. β-actin, is one of the most widely adopted housekeeping genes for gene expression analysis in testicular tissue. Nevertheless, cytoskeletal genes such as β-actin carry a potential risk of expression fluctuation when tissue cellular composition changes, for instance following spermatogenic cell loss triggered by toxicant exposure. To mitigate this concern and enable cross-validation of qPCR results, Hprt1 was introduced as a second independent reference gene. Hprt1 encodes hypoxanthine phosphoribosyltransferase 1, a housekeeping enzyme involved in purine metabolism. Its transcriptional abundance is generally less sensitive to tissue remodeling, cell apoptosis and alterations in cell population that occur in arecoline-injured testes, making it suitable for complementary validation in this testicular toxicology model.

### 4.13. Western Blot

Frozen testicular tissues and GC-1 spg cells were lysed in RIPA lysis buffer (Beyotime, China) supplemented with protease inhibitor cocktail (Roche, Mannheim, Germany), samples were incubated on ice for 10 min, followed by centrifugation, and supernatants were harvested. Following protein quantification, protein samples were separated by SDS-PAGE and electro-transferred onto PVDF membranes (Millipore, Boston, MA, USA). After blocking with 5% non-fat milk, the membranes were then incubated overnight at 4 °C with the following primary antibodies: rabbit anti-Notch1 (Wanleibio, Shenyang, China, diluted 1:1000), rabbit anti-Jagged-1 (Wanleibio, Shenyang, China, diluted 1:1000), rabbit anti-Dll-1 (Wanleibio, Shenyang, China, diluted 1:1000), rabbit anti-BAX (Cohesion Biosciences, Shanghai, China, diluted 1:1000), rabbit anti-Bcl-2 (Cohesion Biosciences, Suzhou, China, diluted 1:1000), rabbit anti-cleaved caspase-3 (Cohesion Biosciences, Suzhou, China, diluted 1:1000), rabbit anti-PCNA (Cohesion Biosciences, Suzhou, China, diluted 1:1000), rabbit anti-Cyclin-D1 (ImmunoWay, Galveston County, TX, USA, diluted 1:1000), and rabbit anti-β-tubulin (ImmunoWay, Galveston County, TX, USA, diluted 1:5000). Subsequently, membranes were incubated with corresponding secondary antibodies for 1 h at room temperature. Immunoreactive bands were visualized using an ECL chemiluminescence system (Millipore Corporation, Billerica, MA, USA) according to the manufacturer’s instructions.

### 4.14. Rescue Experiment

Valproic acid (a Notch1-signaling activator) and FLI-06 (a Notch pathway inhibitor) were purchased from MedChemExpress (Monmouth Junction, NJ, USA). Working concentrations for both compounds were determined based on published literature and preliminary experiments [70,71]. Accordingly, 5 mM valproic acid and 10 μM FLI-06 were selected for 48 h treatment in subsequent cellular assays.

### 4.15. siRNA Transfection

GC-1 cells were seeded in 6-well or 96-well plates. When cells reached 30–40% confluence, Notch1 siRNA and negative control (NC) siRNA (GeneChem Technology, Shanghai, China) were transfected into GC-1 cells using Lipofectamine 3000 Transfection Reagent (Invitrogen) following the manufacturer’s instructions. After 4–6 h of transfection, cells were washed twice with PBS and incubated in fresh complete medium for 24–48 h. The transfection efficiency was validated by qPCR and Western blot assays.

### 4.16. Testicular Oxidative-Stress Biomarker Detection

Frozen rat testicular tissues were homogenized in pre-cooled phosphate-buffered saline. Tissue homogenates were centrifuged, and supernatants were collected for biochemical measurement. Total protein concentration of each supernatant was quantified using the BCA protein assay kit (Beyotime Biotechnology, Shanghai, China). The malondialdehyde (MDA) content and the enzymatic activities of superoxide dismutase (SOD), catalase (CAT), and glutathione peroxidase (GPx) were determined using corresponding commercial assay kits (Beyotime Biotechnology, Shanghai, China), strictly following the manufacturer’s instructions.

### 4.17. Statistical Analysis

Data were analyzed using SPSS version 18.0 (SPSS Inc., Chicago, IL, USA) and presented using GraphPad Prism 5.0 (GraphPad Software Inc., San Diego, CA, USA). The normality of data distribution was assessed using the Shapiro–Wilk test, and homogeneity of variance was evaluated by Levene’s test. When both assumptions were satisfied, one-way analysis of variance (ANOVA) was applied to compare three or more groups, followed by Dunnett’s post hoc test for comparisons of each treatment group against the control. For two-group comparisons, the independent samples *t*-test was adopted. When normality was violated or variances were heterogeneous, the Kruskal–Wallis test followed by Dunn’s post hoc test was used for multi-group comparisons, and the Mann–Whitney U test was used for two-group comparisons. Pearson’s correlation analysis was performed to evaluate the correlations between the expression levels of Notch1, Jagged-1, Dll-1 and testicular morphometric parameters; analyses were based on per-animal values (*n* = 6 rats per group; *n* = 24 for the four-group combined dataset) and bivariate normality was confirmed prior to each test. Where normality was violated, Spearman’s rank correlation was used instead. For CCK-8 proliferation assays with dose (or treatment) and time factors, two-way ANOVA was applied; when the interaction was significant, simple effects were analyzed at each time point by one-way ANOVA, followed by Dunnett’s post hoc test comparing each treatment group against the vehicle control at the corresponding time point. Differences were considered significant at *p* values <0.05 (all figures indicate significant differences at: * *p* < 0.05; ** *p* < 0.01; *** *p* < 0.001). Each experiment was performed in triplicate. Data are presented as mean ± S.D.

## Figures and Tables

**Table 1:** Comparison of tissue weight between male rats exposed to 50 mg/kg, 100 mg/kg, and 200 mg/kg of arecoline and unexposed controls.

Treatment	Control (*n* = 6)	50 mg/kg (*n* = 6)	100 mg/kg (*n* = 6)	200 mg/kg (*n* = 6)
Body weight (g)	315.27 ± 20.42 ^a^	292.15 ± 22.17	293.13 ± 16.86	268.06 ± 19.51 **
Testes (g)	3.11 ± 0.91	2.70 ± 0.28	2.85 ± 0.13	2.78 ± 0.22

N: number of male rats. ^a^ Mean ± standard deviation. ** *p* < 0.01, compared with control group (*n* = 6).

**Table 2:** **Primers for quantitative real-time polymerase chain reaction (qRT-PCR) analysis.**

Genes	Forward Primer (5′-3′)	Reverse Primer (5′-3′)
**Notch1**	TCCACCAGTTTGAATGGTCAAT	CGCAGAGGGTTGTATTGGTTC
**Jagged-1**	ATTACCAGGATAACTGTGCGAA	CAAATGTGCTCCGTAGTAAGAC
**Dll-1**	AGCAAGCGTGACACCAAGTG	TTTCAGATGCTTCTCCACCCCT
**BAX**	TCAGGATGCGTCCACCAAGAAG	TGTGTCCACGGCGGCAATCATC
**Bcl-2**	ATCGCCCTGTGGATGACTGAGT	GCCAGGAGAAATCAAACAGAGGC
**caspase-3**	TACTCCTTCCATCAAATAGAACCAC	TCATAATGACTGCACCAGTATCCA
**PCNA**	CAAGTAATGTCGATAAAGAGGAGG	GTGTCACCGTTGAAGAGAGTGG
**Cyclin D1**	TAGGTTCCATCCTTTACGTG	TACAGAGATGCCTAGAACCC
**β-actin**	GAGGGAAATCGTGCGTGAC	GCATCGGAACCGCTCATT
**Hprt1**	CTCATGGACTGATTATGGACAGGAC	GCAGGTCAGCAAAGAACTTATAGCC

## Data Availability

The data generated in this study are available from the corresponding author upon reasonable request.

## Supplementary Materials

The following supporting information can be downloaded at: https://www.mdpi.com/article/10.3390/ijms27219728/s1.
